# Evaluation of Dental Arch Space Changes and the Need for a Space Maintainer After Unilateral Loss of Maxillary First Primary Molar: A Systematic Review and Meta-Analysis

**DOI:** 10.7759/cureus.81073

**Published:** 2025-03-24

**Authors:** Vidhi J Joshi, Chhaya Patel, Megha C Patel, Miyola C Fernandes, Disha Makwani, Foram Patel

**Affiliations:** 1 Department of Pedodontics and Preventive Dentistry, Karnavati School of Dentistry, Karnavati University, Gandhinagar, IND; 2 Dentistry, Gold Dental Studio, Panvelim, IND; 3 Public Health, Asian Development Research Institute, Patna, IND

**Keywords:** premature tooth loss, primary maxillary first molar, space loss, space maintainer, spatial changes

## Abstract

Space changes due to early exfoliation of primary teeth in mixed dentition have been an important aspect in pediatric dentistry as it can impact the dental growth of an individual. This systematic review aimed at examining the spatial changes in dental arches caused by the early loss of maxillary first primary molars and to evaluate whether a space maintainer was necessary in such cases. This review was registered at PROSPERO (International Prospective Register of Systematic Reviews) database (registration number: CRD42024566890) and performed according to the Preferred Reporting Items for Systematic Reviews and Meta-Analysis guidelines. A search was done electronically for articles published in English from 2000 to 2023. In total, seven studies with the split mouth design involving early extraction of a maxillary deciduous first molar unilaterally were included. STATA version 17.0 (StataCorp LLC, College Stattion, TX, USA) was used to assess the random effects model, heterogeneity, and publication bias. Arch perimeter, arch length, arch width, intercanine width, and intercanine length were evaluated, along with the space alterations of the first and second molar (D + E) spaces before and following extraction. Significant space loss was observed between the control and extraction side (p = 0.02) at six months post-extraction. Other parameters such as the intercanine length (p = 0.01), intercanine width (p = 0.04), and arch perimeter (p = 0.04) also showed increased significant values at 8-10 months follow-up. Following the premature loss of the maxillary first primary molar, space loss occurred but did not significantly affect the arch perimeter, intercanine width, arch width, or length. Factors such as time elapsed since extraction, dental arch crowding, age of the patient, facial pattern, and intermolar relationships affected the treatment plan.

## Introduction and background

Exfoliation of a tooth that occurs 12 months or more before the typical time for permanent tooth eruption and surpasses the typical variability in exfoliation sequences is considered premature loss of deciduous teeth [1]. Several environmental and morphogenic factors govern the occlusal relationship, and any disruption in one of these elements may have a negative impact on occlusion. Because of their morphology, children’s poor eating habits, and inappropriate feeding techniques, primary teeth are frequently linked to dental caries. Dental decay is the most frequent cause of early exfoliation of primary teeth. Trauma, ectopic eruption, congenital conditions, and abnormalities in arch length that result in the resorption of primary teeth are other reasons for premature tooth loss [2].

The consequences of early tooth loss are multifaceted and include both morphological and functional aspects. Clinically, the consequences rely on several variables, including the number, topography, and edentulous forms of extracted teeth. Whether the edentulous teeth are frontal or lateral, symmetric or asymmetric, mono-maxillary or bi-maxillary, isolated or continuous should be observed. Determining the pace of exfoliation of the primary teeth relative to the sequence of permanent teeth eruption is also an important aspect. Thus, premature loss of the second primary molar ultimately leading to premature eruption of the second premolar results in loss of available space, which is necessary to align the canines and premolars. The pre-existence of vicious habits or malocclusions is important as that may lead to irreversible and more severe manifestations in relation to the edentulous space [3]. Super-eruption of the opposite arch tooth and mesial and distal drifting of the teeth adjacent to the lost tooth are all possible outcomes of early tooth loss.

The American Academy of Pediatric Dentistry 2021 guidelines state that the objectives of space maintenance are to prevent the loss of arch length, arch width, and/or arch perimeter by maintaining the relative position of the existing dentition [4]. It is well acknowledged that, depending on the kind and timing of tooth loss, a malocclusion will result from a break in the arch integrity of the primary or mixed dentition without the assistance of a device such as a space maintainer. The safest way to retain space in the dental arch is to place a space maintainer in patients whose primary teeth must be extracted for significant caries or other reasons. In a study of 225 schoolchildren, Miyamoto and colleagues showed that, when no space maintenance was used, the early loss of primary canines and molars necessitated orthodontic treatment [5].

Although a space maintainer is typically recommended following the exfoliation of the primary second molar, there is much disagreement on the necessity of space maintainers and space management following the premature loss of the primary first molar. The majority of research has demonstrated that the drift of permanent incisors and deciduous canine teeth toward the edentulous areas in both arches is linked to the loss of space in the first four to six months following extraction.

Although mesial drifting of the primary second molar into the extraction area is more common in the maxilla, generally, early loss of the primary mandibular first molar primarily causes distal drifting of the primary mandibular canine [6]. Mesial forces may cause the deciduous first molar space to be lost during the vigorous eruption of permanent first molars, which occurs between the ages of five and seven years. Antagonistic teeth may emerge as a result of unilateral maxillary early loss of deciduous molars, which might obstruct mandibular dynamics. Premature loss of primary maxillary molars results in anterior cross-bite and palatal tilting of the permanent maxillary incisors. Unilateral cross-bite, collapsed deep bite, and inadequate alveolar local development with asymmetrical upper endo-alveoli are caused by lingual tilting, over-positioning of the mandibular permanent incisors, and functional mandibular prognathism [7].

The consequences of space modifications after primary molar loss were evaluated in earlier systematic reviews by Gandhi and Gurunathan [2] and Zhao et al. [1]. The impacts on space loss caused by any primary tooth, whether maxillary or mandibular, first or second molar, were examined in the systematic review by Gandhi and Gurunathan [2]. Zhao et al. [1] used variables such as arch width, arch length, and arch perimeter changes to examine the impact of both maxillary and mandibular first molar loss on space loss. While some suggested that the first molar did not significantly alter the length of the arch, Tunison et al. [4] and Lin and Chang [8] felt that a space maintainer was necessary for the premature loss of the primary maxillary first molar. There is a dearth of literature on evaluation including every potential element with an impact on the specifics of loss of space after missing deciduous first molars during the early stage of mixed dentition. To avoid the needless placement of space maintainers, this study sought to thoroughly investigate all relevant parameters and determine if any intervention such as a space-holding device was necessary in cases of a missing deciduous maxillary first molar.

## Review

Methodology

Study Protocol

The registration of this review was done in PROSPERO (International Prospective Register of Systematic Reviews) database (registration number: CRD42024566890) and was conducted according to the Preferred Reporting Items for Systematic Reviews and Meta-Analyses (PRISMA) guidelines. Adhering to the PRISMA guidelines facilitated the rigorous selection and evaluation of studies, standardized data extraction, and transparent reporting of the findings. By following the PRISMA protocol [9], this study enhanced the reliability, credibility, and reproducibility of the statistical findings obtained through this review.

Eligibility Criteria

The selection of articles was done based on the inclusion and exclusion criteria as listed in Table 1.

**Table 1:** Eligibility criteria.

Inclusion criteria	Exclusion criteria
Studies included patients having unilateral loss of maxillary primary first molars	Studies with both maxillary deciduous molars missing, mandibular first molars, or even missing maxillary second molars were excluded
Articles in which the age range of patients was 5–10 years	Systematic reviews, retrospective and prospective clinical studies, reviews, surveys, editorials, and letters to the editor were excluded
Articles with patients with missing first molar and without any appliance or space maintenance device	Studies that included patients with malocclusions, confirmed the congenital absence of tooth/teeth, or the presence of supernumerary teeth and growth disorders were not considered for this study
Non-randomized clinical trials with split-mouth designs	-
Articles in only the English language	-
In the case of multiple publications, the most recently published article was considered	-

Research Question

The PECOS [10] format presented in Table 2 was used to formulate the review questions.

**Table 2:** PECOS format.

Criteria	Determinants
P (Population)	Patients with a missing maxillary deciduous first molar
E (Exposure)	Extraction of maxillary deciduous first molar unilaterally
C (Comparison)	Comparison between the extraction side and the contralateral side with no maxillary molar missing
O (Outcome)	Modifications in the spatial configuration of dental arch and a need for space maintainer
S (Study design)	Split-mouth studies were extracted for this review

The PECOS question explored was “Are the dental arch spatial changes after the premature loss of maxillary primary first molar similar to the side with no loss?”

Literature Search

English-language articles published on PubMed, EBSCO, and Google Scholar were screened from January 2000 to December 2023. Table 3 presents an overview of the search process. The investigational methodology employed encompassed a synthesis of MeSH terminology and pivotal keywords such as “primary maxillary first molar,” “tooth migration,” “distal movement,” “mesial movement,” “space loss,” “premature tooth loss,” and “space maintainer.” These keywords were combined with Boolean operators “AND”/“OR” with an advanced search in addition to the MeSH terms generated by PubMed to obtain the most suitable results. To find the pertinent publications, review articles and references from other studies were also used.

**Table 3:** Search strategy.

Database	Keywords	Number of articles
PubMed	(primary maxillary first molar) AND (((tooth drift) OR (tooth migration) OR (distal movement) OR (mesial movement) OR (space loss) OR (arch changes)))) AND (((premature loss) OR (premature tooth loss) OR (tooth extraction) OR (tooth loss) OR (tooth exfoliation) OR (space maintainer)))	56
Google Scholar	(primary maxillary first molar) AND (((tooth drift) OR (tooth migration) OR (distal movement) OR (mesial movement) OR (space loss) OR (arch changes)))) AND (((premature loss) OR (premature tooth loss) OR (tooth extraction) OR (tooth loss) OR (tooth exfoliation) OR (space maintainer)))	6,900
EBSCO	(primary maxillary first molar) AND (((tooth drift) OR (tooth migration) OR (distal movement) OR (mesial movement) OR (space loss) OR (arch changes)))) AND (((premature loss) OR (premature tooth loss) OR (tooth extraction) OR (tooth loss) OR (tooth exfoliation) OR (space maintainer))))	1

Data Extraction

We retrieved pertinent information from each of the included studies (Table 4). Study details such as authors and publication year, details of the methodology (study design), follow-up period, and the form in which data was collected (e.g., plaster casts) were recorded. Details of the participants included age range, number of participants, and arch with tooth loss. D + E space loss was the primary evaluation indicator, while arch perimeter, arch length, intercanine width, intercanine length, and arch width were the secondary evaluation parameters that were measured with model analysis. The type of molar and canine relationship, arch perimeter, and midline deviation were studied wherever applicable in select studies.

**Table 4:** Data extraction table.

Study detail	Study design	Age (in years)	Sample size	Data collection	Follow-up (in months)	Arch	Evaluation indicators	D + E Space loss (in mm)
Mosharrafian et al. (2021) [6]	Cross-sectional, split-mouth	6–8	25	Plaster casts	6–24	Maxilla	Type of molar and canine relationship, facial pattern, midline deviation, space loss	1.32
Heidari et al. (2022) [11]	Cross-sectional, split-mouth	8–10	25	Plaster casts	6–24	Maxilla	Midline deviation, molar and canine relationships at both sides, facial growth pattern, and the amount of space loss	0.54
Kobylińska et al. (2019) [12]	Longitudinal study, split-mouth	5–7	16	Plaster casts	1, 3, 6, 12	Maxilla	Circumference of the arch, the posterior and intercanine width, as well as the interdental distance on the side of extraction and the opposite side	1.156
Lin et al. (2011) [13]	Longitudinal study, split-mouth	6–9 6.0 ± 0.74)	13	Plaster casts	12	Maxilla	Arch width, arch length, intercanine width, intercanine length, arch perimeter, space loss	0.82
Lin et al. (2017) [14]	Longitudinal study, split-mouth	5–7	9	Plaster casts	81	Maxilla	Arch width, arch length, intercanine width, intercanine length, and arch perimeter, space loss	Not mentioned
Lin et al. (2007) [15]	Longitudinal study, split-mouth	4–7	19	Plaster casts	6	Maxilla	Arch width, arch length, intercanine width, intercanine length, and arch perimeter, space loss	1.08 ± 0.44
Park et al. (2009) [16]	Cross-sectional, split-mouth	6–10	13	Digitized plaster casts	12	Maxilla	Primary molar space, arch width, arch length, and arch perimeter	0.57 ± 0.83

Statistical Analysis

When the quantity and quality of the data supported it, a meta-analysis was performed. Heterogeneity, publication bias, and the random effects model were examined using Stata software version 17 (StataCorp LLC, College Station, TX, USA). Mean differences (MDs) were used to determine whether the differences in spaces between baseline and final assessment values were statistically significant. Heterogeneity was assessed using I^2^ statistics and Cochrane’s Q test, with I^2^ > 50% or p < 0.10 on Cochrane’s Q test indicating substantial heterogeneity [17]. Statistical significance was operationally characterized by a p-value threshold of less than 0.05. Publication bias was evaluated by visual inspection of the funnel plot [18], as well as the fail-safe N test. Additionally, sensitivity analysis and Begg’s and Egger’s test results were examined using Stata software.

Results

**Figure 1:**
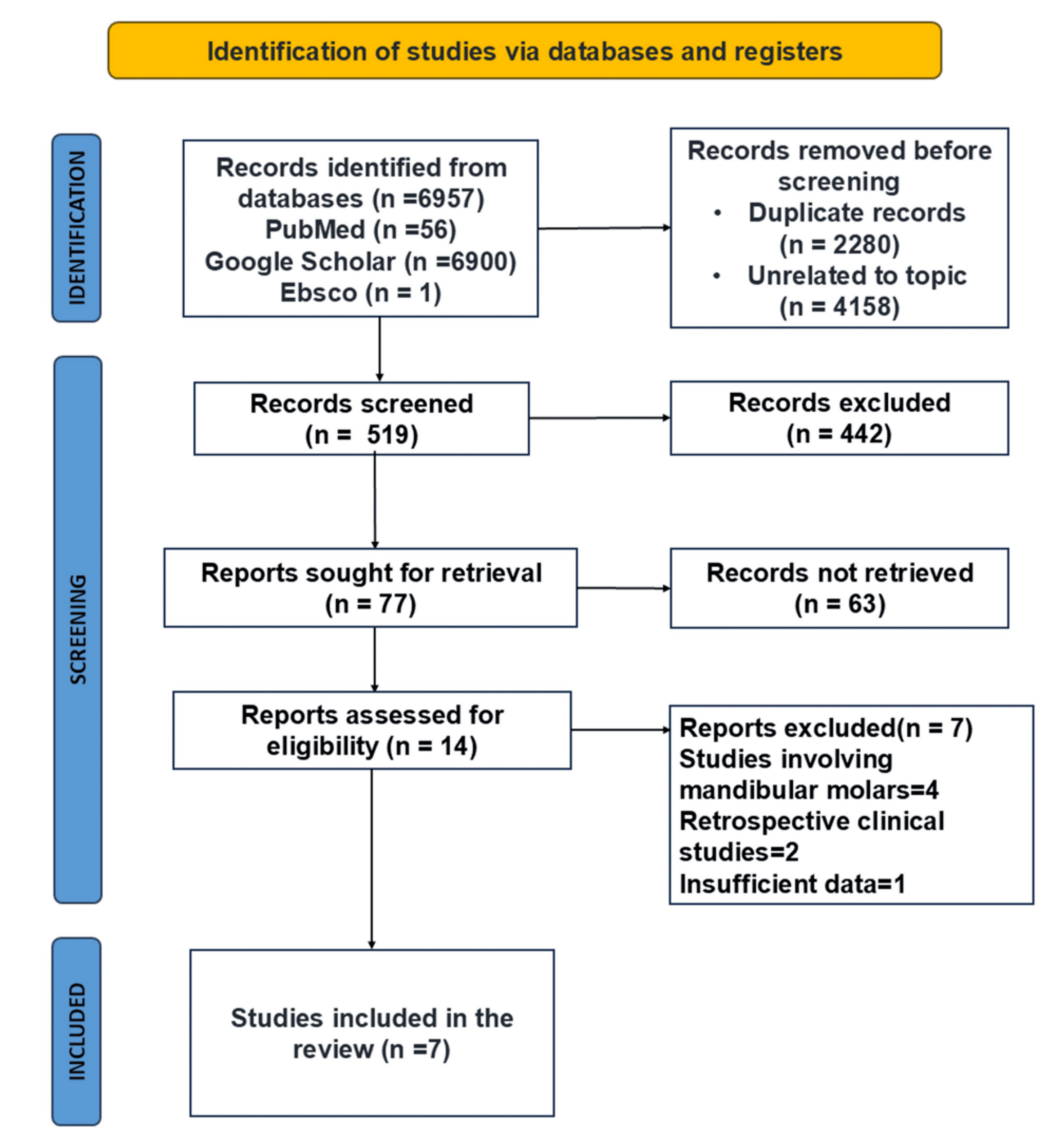
Preferred Reporting Items for Systematic Reviews and Meta-Analysis flowchart. This figure was created by Vidhi Joshi

Based on the electronic search in the three databases, a total of 6,957 articles were identified. The screening was done based on the titles, abstracts, and eligibility criteria of the review, and, finally, seven studies [11-16] that met the inclusion criteria, including 120 patients, were included in the systematic review, as shown in the PRISMA flowchart (Figure 1). The average age range of the patients included was 5-10 years in all the studies included. The first and second molar D + E space, intercanine width, intercanine length, arch width, arch length, and arch perimeter were the metrics used to measure the spatial differences between the intervention and control sides.

Quality Appraisal

A supervisor analyzed the results obtained from the selected studies and assessed the methodological quality of the results. A single author also assessed the possibility of bias. The Newcastle-Ottawa scale [19] was used for the assessment of risk of bias for non-randomized clinical trials. Each study was evaluated based on all three domains scoring the reliability of the study based on various factors (Table 5). Based on the total scores of all the domains, the risk of bias was calculated to be good, fair, or poor. If the total score obtained was 0-2, they were rated as poor, 3-5 was rated as fair quality, and 7-9 was rated as good/high quality.

**Table 5:** Assessment of the risk of bias of each included study using the Newcastle-Ottawa scale.

Study	Selection				Comparability	Outcome			Total quality score
	Representativeness of the exposed cohort	Selection of the non-exposed cohort	Ascertainment of exposure	Demonstration that outcome of interest was not present at start of study	Comparability of the cohorts based on the design or analysis controlled for confounders	Assessment of outcome	Was follow-up long enough for outcomes to occur	Adequacy of follow-up of cohorts	
Mosharrafian et al. (2021) [6]	1	0	1	1	1	0	1	1	6
Heidari et al. (2022) [11]	1	0	1	1	1	0	1	1	6
Kobylińska et al. (2019) [12]	1	1	1	1	1	1	1	1	8
Lin et al. (2011) [13]	1	1	1	1	1	1	1	1	8
Lin et al. (2017) [14]	0	0	1	1	1	0	1	1	5
Lin et al. (2007) [15]	1	0	1	1	1	0	1	1	6
Park et al. (2009) [16]	1	1	1	1	1	1	1	1	8

Of the studies included, three were cross-sectional studies [6,11,16] and four were longitudinal studies [12-15]. A split-mouth technique was employed in all studies to avoid any variability in terms of treatment effects that may have been present interindividually. A total of 120 participants were included in the study, all of whom were involved in the split-mouth design and had unilateral premature maxillary first molar loss. All studies [6,11,16] measured the space loss (D + E) on the control side as well as the extraction site. Of the total included studies, plaster casts were used for data collection in six studies [6,14-19], and digitized plaster casts were used in one study [19].

Space Loss

Heidari et al. (2022) [11] noted the space loss in the maxilla to be 0.54 mm (p > 0.05). Kobylińska et al. (2019) [12] found significant space loss (p < 0.05) at a 12-month follow-up. Lin et al. (2007) [15] (p = 0.01), Lin et al. (2011) [13] (p = 0.02), and Lin et al. (2017) [14] (p < 0.05) found significant space loss on the extraction side compared to the control side at an average of 6-81-month follow-up.

Arch Width

Lin et al. (2007) [15] (p = 0.204) and Lin et al. 2011 [13] (p = 0.757) found no significant difference in arch width whereas a significant increase was observed at the initial and 81-month follow-up by Lin et al. (2017) [14] (p = 0.023). Park et al. [16] reported a 0.54 mm increase in the arch width at a 12-month follow-up.

Arch Length

Lin et al. (2017) [14] (p = 0.007) observed a significant increase in arch length at the 81-month follow-up whereas Lin et al. (2011) [13] (p = 0.081) noted an increase at the 12-month follow-up after extraction. Kobylińska et al. 2019 [12] reported that the mean maxillary anterior arch length after 12 months was lower compared to baseline. Park et al. [16] found out an increase of 1.1 mm at a 12-month follow-up.

Arch Perimeter

Lin et al. (2017) [14] (p = 0.071) and Lin et al. (2007) [15] (p = 0.246) did not observe a significant increase in arch perimeter, whereas Lin et al. (2011) [13] (p = 0.043) concluded a significant increase. Kobylińska et al. [12] concluded that the mean dental arch perimeter increased by 1.44 mm (p > 0.05) in the maxilla within a year. Park et al. [16] reported an increase of 1.84 mm from the initial value at a 12-month follow-up.

Intercanine Length

Lin et al. (2007) [15] found no significant change while Lin et al. (2011) [13] (p = 0.001) and Lin et al. (2017) [14] (p = 0.002) observed a significant increase at the 12 and 81-month follow-up, respectively.

Intercanine Width

Lin et al. (2007) [15] (p = 0.001), Lin et al. (2011) [13] (p = 0.00), and Lin et al. (2017) [14] noted a significant increase in intercanine width at a follow-up of 6 months and 12 months, respectively. Kobylińska et al. [12] observed that the mean baseline intercanine width was 33.69 (±4.42) which showed an increase at the 12-month follow-up (p > 0.05).

Meta-analysis for the primary outcome

**Figure 2:**
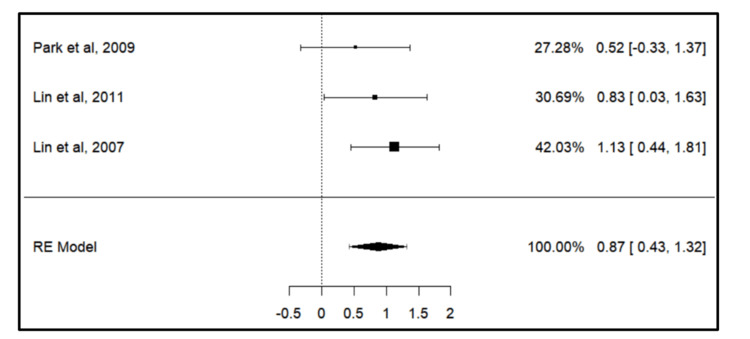
Forest plot showing space changes between the control and extraction side. Park et al. [16], Lin et al. (2011) [13], and Lin et al. (2007) [15] RE model: random effect model This image was created by Vidhi Joshi

A total of three empirical investigations were incorporated into the meta-analytical assessment of spatial loss. The documented standardized mean differences varied between 0.5225 and 1.1294, with a predominant proportion of estimates exhibiting positive values (100%). According to the Q-test, there was no significant heterogeneity in the true outcomes (Q(2) = 1.2020, p = 0.5483, tau² = 0.0000, I² = 0.0000%). The forest plot (Figure 2) showed a positive correlation between space loss on the extraction side of the arch. The effect size of the study by Lin et al. (2007) [15] was the maximum, hence showing significant results.

Meta-analysis for the secondary outcomes

**Figure 3:**
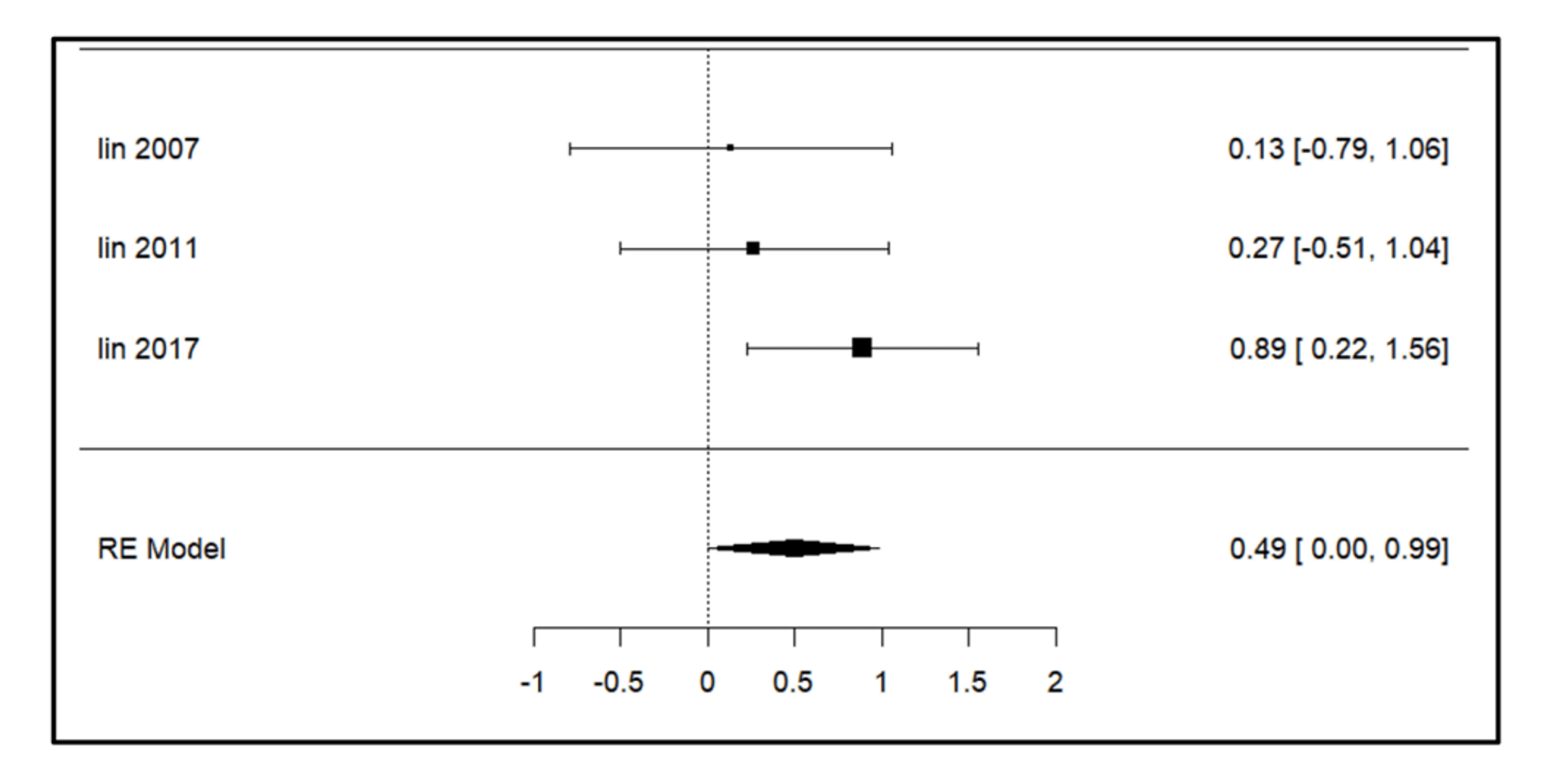
Forest plot showing changes in arch perimeter. Lin et al. (2007) [15], Lin et al. (2011) [13], and Lin et al. (2017) [14] RE model: random effect model This image was created by Vidhi Joshi

**Figure 4:**
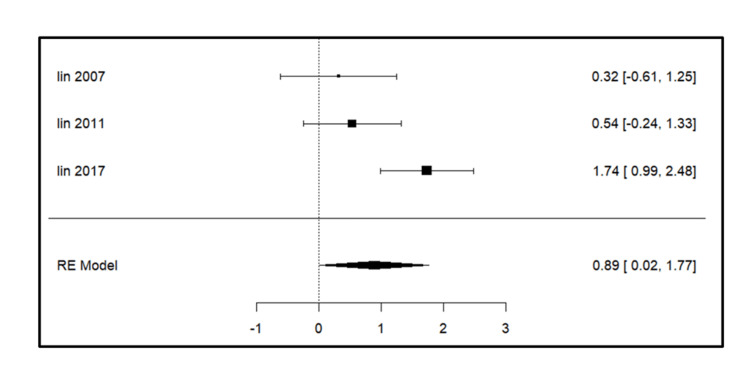
Forest plot showing changes in intercanine width. Lin et al. (2007) [15], Lin et al. (2011) [13], and Lin et al. (2017) [14] RE model: random effect model This image was created by Vidhi Joshi

**Figure 5:**
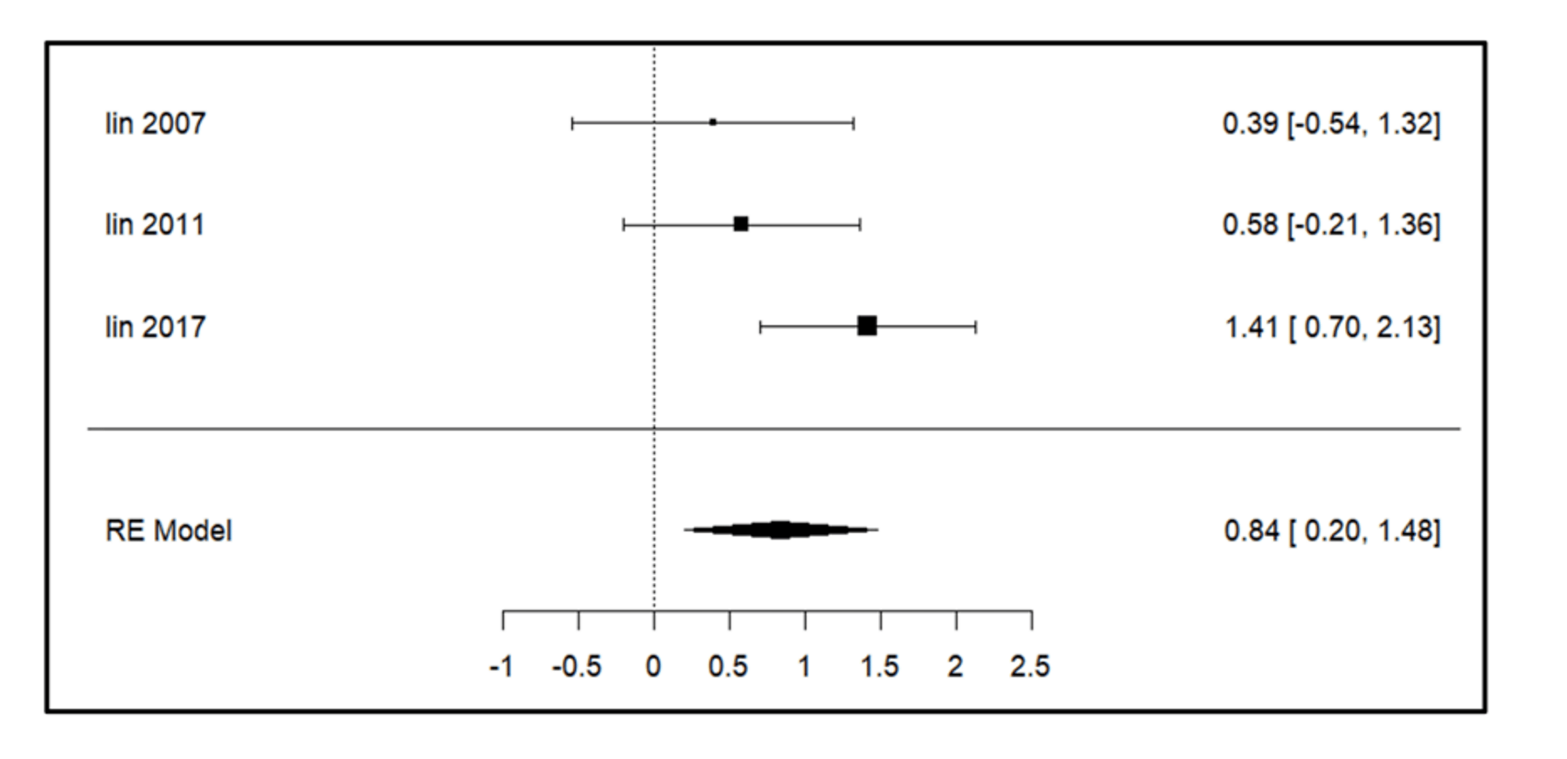
Forest plot showing changes in intercanine length. Lin et al. (2007) [15], Lin et al. (2011) [13], and Lin et al. (2017) [14] RE model: random effect model This image was created by Vidhi Joshi

**Figure 6:**
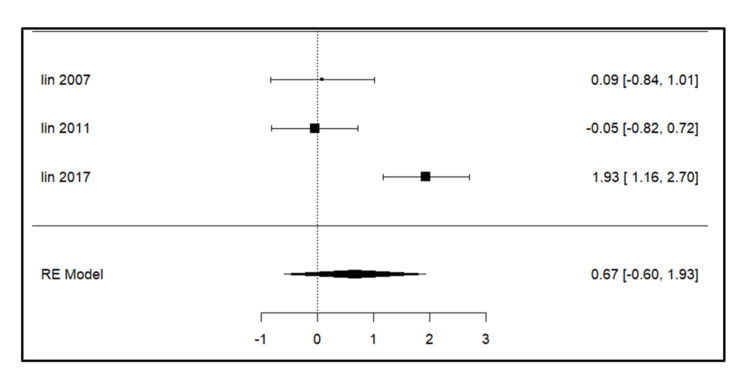
Forest plot showing changes in arch width. Lin et al. (2007) [15], Lin et al. (2011) [13], Lin et al. (2017) [14] RE model: random effect model This image was created by Vidhi Joshi

**Figure 7:**
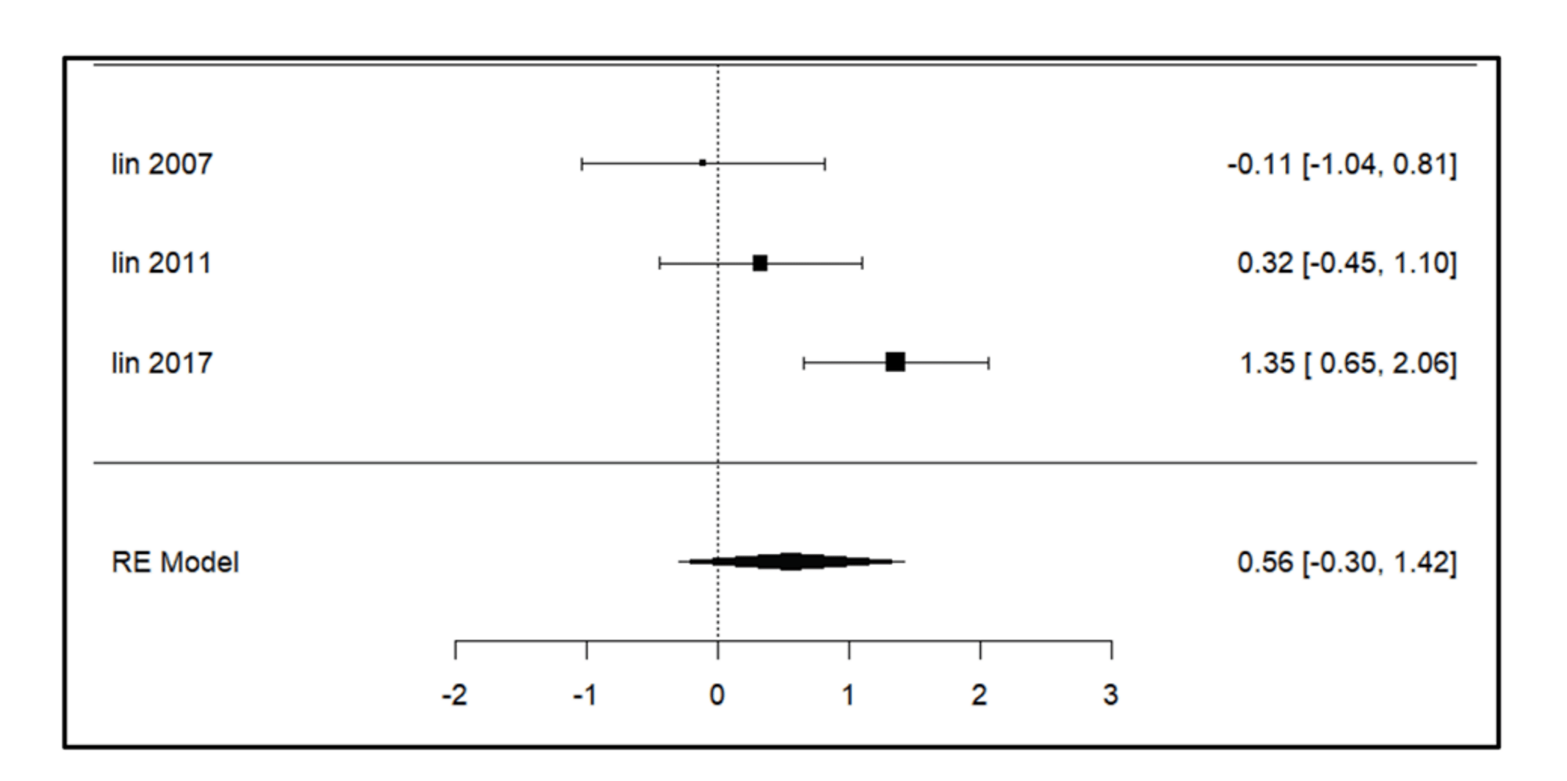
Forest plot showing changes in arch length. Lin et al. (2007) [15], Lin et al. (2011) [13], and Lin et al. (2017) [14] RE model: random effect model This image was created by Vidhi Joshi

A total of three studies were included in the analysis for parameters such as intercanine width, intercanine length, arch width, arch perimeter (Figure 3), and arch length. For intercanine width (Figure 4), there was a significant increase on the extraction side in the study by Lin et al. (2017) [14]. The intercanine length (Figure 5) values were increased in the follow-up period most significantly in the study done by Lin et al. (2017) [14]. The forest plot analysis for arch width (Figure 6) and arch length (Figure 7) showed no significant change on the extraction and control side at a follow-up period of 6-24 months.

Discussion

The systematic review examines the changes in space after the early loss of primary maxillary first molars. According to McDonald and Avery [20], the loss of both maxillary and mandibular primary first molars typically leads to similar space loss, though the degree of loss often depends on the time elapsed since extraction. There is still much disagreement on the need for space maintenance and efficient clinical care following the early loss of these molars.

This review aimed to evaluate not only overall space changes but also specific metrics, such as arch width and intercanine width, involving the premature loss of primary maxillary first molars. It is important to note that most studies included in the review had follow-up periods of less than 24 months, except Lin et al. (2017) [14], which had an extended follow-up of 81 months. This variation in the follow-up duration may affect the reliability of outcomes reported in shorter studies, underscoring the need for further research to assess long-term impact.

D + E Space Loss

According to Lin et al. (2011) [13], there was significant space loss D + E on the extraction side compared to the control side with no apparent significant increase in the arch width or length. This indicates the space loss is a result of the distal movement of the primary canine in the extraction space. The space loss in the maxillary first and second primary molar (D + E) was 0.65 mm, similar to the Heidari et al. study [11] (0.54 mm). In the study by Lin et al. (2007) [15], the first and second molar space was significantly reduced along with a reduction in arch length and an increase in intercanine width, indicating the space loss to be due to the distal movement of the primary canine and incisors. Kobylińska et al. [12] found that the loss of space was due to the distalization of the primary canine and mesialization of the second primary molar on both the maxillary and mandibular arches. According to the Kronfeld theory, the teeth posterior to the neutral areas drift mesially, and the teeth anterior to the neutral areas drift distally. Padma and Retnakumari [21] observed the greatest space loss in the first four months after premature extraction, but space loss subsequently increased gradually and became stable over 6-24 months.

Arch Width and Arch Length

There were no statistically significant differences due to the loss of the primary molar at the one-year follow-up, which suggests that distalization of canine and second primary molar mesialization are the main factors for space loss, as supported by Kobylińska et al. [12], Padma and Retnakumari et al. [21], Macena et al. [22], and Lin et al. (2011) [13]. On the other hand, Lin et al. (2017) [14] derived a significant increase in these parameters at the 81-month follow-up. Lin et al. (2007) [15] found a significant decrease (p = 0.014) at a six-month follow-up, which was attributed to distal drifting of the incisors and primary canines in the extraction space.

Arch Perimeter

Lin et al. (2017) [14] (p = 0.071) and Lin et al. (2007) [15] (p = 0.246) did not observe a significant increase in arch perimeter, whereas Lin et al. (2011) [13] (p = 0.043) concluded a significant increase. The significant rise in intercanine length and width suggested that the canines and permanent incisors erupted in a labial position, increasing the overall arch dimension. Kobylińska et al. [12] reported an increase in the arch perimeter at a one-year follow-up (p > 0.05).

Intercanine Width and Intercanine Length

Lin et al. (2011) [13] noted an increase in arch length at the 12-month follow-up but not at six months, suggesting that the increased arch dimension was gained during the second six-month follow-up, especially in the anterior segment (intercanine width and intercanine length). Lin et al. (2007) [15] found a significant increase at the six-month follow-up mainly due to the distal drifting of the anterior teeth on the extraction side. No statistically significant increase was seen at a 12-month follow-up on the extraction side, as stated by Kobylińska et al. [12].

Facial Pattern

Alexander et al. [5] and Mosharrafian et al. [6] showed greater space loss in children with hyperdivergent facial patterns compared with hypodivergent patients. Alexander et al. [5] showed that the facial growth pattern in children >7 years of age, similar to molar occlusion patterns, can affect the need for a space maintainer when the primary first molar is lost prematurely. In the patients with facial form mesoprosopic (mean value of 1.84 mm) and euryprosopic (mean value of 0.94 mm) groups, the mean space loss was lower. According to the results of the linear regression analysis, the face pattern had a substantial impact on the amount of space loss, that is, the space loss significantly decreased as the ratio of facial width to height increased. Heidari et al. [11] stated that the difference between the leptoprosopic and euryprosopic patterns was significant (p = 0.048).

Age and Time Since Extraction

According to Heidari et al. (2022) [11], the first six months after extraction are the most crucial for space loss. Mosharrafian et al. [6] reported that the maximum space loss at six to nine months from extraction, which might be from the specific age group where the transition during mixed dentition can occupy the available space. According to Heidari et al. [11], as people age, their bone density increases, making it harder to move their teeth. Terlaje and Donly [23] reviewed the treatment plans for space maintenance and reported that no treatment was administered for the unilateral loss of a primary first molar in patients in whom the permanent first molar had erupted unless the leeway space was to be preserved. They argued that the erupted molars are passive, thereby not producing a mesial component of eruption force.

Limitations

This review had a few limitations. Only three articles were considered in the meta-analysis of first molar space loss which cannot be standardized due to low reliability. Additionally, the follow-up periods varied among the included studies, with the majority being fewer than 24 months. As a result, it could be required to prolong the follow-up periods until the succedaneous permanent teeth erupt.

## Conclusions

A space maintainer is a valuable tool in cases where there is premature loss of the primary teeth to prevent any major malalignment of the successor teeth. However, the applications of a space maintainer are to be carefully evaluated, especially in cases when the primary maxillary first molar is lost. After premature loss of the first primary molars, space can be lost, but the amount of loss would not clinically affect arch width, length, or arch perimeter over the 6-24-month follow-up period. Premature loss of first primary molars results in interdental distances reduced to an extent that does not interfere with the eruption of a permanent successor. Factors such as age, time since tooth extraction, facial pattern, and molar relationships also influenced the space change after the premature loss of the first primary molar. It is suggested to precisely assess these related factors to decide whether to place a space maintainer for a prematurely lost primary first molar. Based on the above-stated observations, it can be said that a space maintainer is questionable, especially where the permanent first molar has already erupted. However, in cases of mild anterior crowding, a space maintainer could preserve the leeway space which will be utilized later to resolve the crowding issues.
